# Identification of Differential Metabolites in Chronic Suppurative Otitis Media With Non‐Targeted and Targeted Metabolomics Approach

**DOI:** 10.1002/smmd.70015

**Published:** 2025-07-30

**Authors:** Lidan Hu, Yifan Zhu, Chengpeng Wu, Xiao Liu, Qi Wang, Yangyiyi Huang, Hongyan Liu, Xiangjun Chen, Wei Wu, Hua Jiang

**Affiliations:** ^1^ Department of Otolaryngology The Second Affiliated Hospital Zhejiang University School of Medicine Hangzhou China; ^2^ Children's Hospital Zhejiang University School of Medicine National Clinical Research Center for Child Health Hangzhou China; ^3^ Eye Center of the Second Affiliated Hospital Zhejiang University School of Medicine Hangzhou China

**Keywords:** bone erosion, chronic suppurative otitis media, metabolomics, middle ear cholesteatoma

## Abstract

Chronic suppurative otitis media (CSOM) is a leading cause of hearing loss and otorrhea, and when associated with cholesteatoma, it can pose a serious threat to patients' lives. This study aims to identify differences in tissue metabolites between patients with CSOM, both with and without cholesteatoma. Metabolomic profiles were measured in tissue samples from 42 surgically treated CSOM patients (35 with cholesteatoma, 7 without cholesteatoma). Significantly altered metabolites associated with CSOM were identified using a non‐targeted metabolomics approach and a targeted metabolomics approach. The 42 patients were divided into screening and validation sets. The non‐targeted analysis revealed 484 distinct differential metabolites and 32 metabolic pathways that differed between CSOM with and without cholesteatoma in the screening set. Targeted metabolomics confirmed that levels of azobenzene and marimastat in the validation set exhibited trends similar to those observed in the non‐targeted analysis. Azobenzene and marimastat were found to be associated with the differences between CSOM with and without cholesteatoma, as well as with bone erosion in the middle ear. This study identified novel potential metabolic pathways and metabolites, providing insights into their possible roles in the inflammatory processes and bone erosion associated with CSOM and cholesteatoma.

## Key Points

- The integration of non‐targeted and targeted metabolomics identifies stage‐specific metabolic profiles distinguishing chronic suppurative otitis media and cholesteatoma progression, validated across independent cohorts.
- Inflammation‐mediated bone erosion is linked to enriched metabolic pathways, revealing novel mechanisms for immune dysregulation and oxidative stress during cholesteatoma progression.

## Graphical abstract

Untargeted metabolomics is used to analyze tissue samples from chronic suppurative otitis media patients with or without cholesteatoma and identify metabolic differences across groups stratified by CT scores. Targeted metabolomic validation in an independent cohort verified five candidate metabolites.

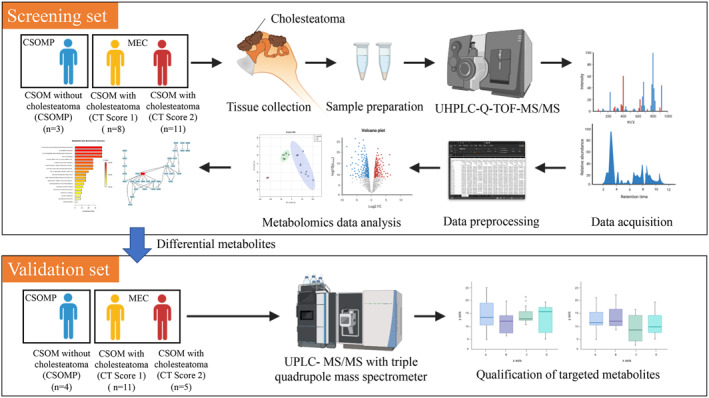

**Abbreviations**
AUC: area under the curve
CI: confidence interval
CSOM: chronic suppurative otitis media
CV: coefficients of variation
ESI: electrospray ionization
FDR: false discovery rate
KEGG: kyoto encyclopedia of genes and genomes
MEC: middle ear cholesteatoma
MMP: matrix metalloproteinase
OPLS‐DA: orthogonal projections to latent structures discriminant analysis
PCA: principal component analysis
ROC: receiver operating characteristic curve
UHPLC‐QTOF‐MS: ultra‐high performance liquid chromatography‐quadrupole time of flight‐mass spectrometry
UPLC‐MS/MS: ultraperformance liquid chromatography‐tandem mass spectrometry

## 1 Introduction

Chronic suppurative otitis media (CSOM) is a persistent or recurrent infection of middle ear [1, 2], leading to hearing loss, otorrhea, and a significant decline in health‐related quality of life [3, 4]. CSOM affects 65–330 million people globally, with 60% experiencing hearing loss [5]. Early diagnosis and effective treatment for CSOM are primary clinical objectives for Otolaryngologists. The broad classification of CSOM includes two common types: CSOM without cholesteatoma and CSOM with cholesteatoma [1, 6]. In CSOM patients, otoscopy images and high‐resolution temporal bone CT scans are crucial for diagnosis and treatment planning, especially in cases where “unsafe ear” with cholesteatoma is suspected [2].

Middle ear cholesteatoma (MEC) is a destructive lesion that locally invades the middle ear and comprises congenital and acquired types [7, 8]. The acquired form has an annual incidence of 7 and 15 per 100,000 individuals [7], with a higher prevalence in adults [9]. Acquired MEC usually arises from eustachian tube dysfunction or CSOM (in about one‐fourth of cases) [10, 11], leading to severe bone erosion from the ossicular chain to the bony labyrinth [12], which can result in progressive deafness, facial paralysis, and other life‐threatening intracranial complications [13]. Bone erosion has been treated as the primary mechanism of cholesteatoma invasion [14], and while how it progresses has been extensively studied at the cellular level and signaling pathways [15, 16, 17, 18], dynamic changes in metabolites during these pathological processes remain poorly understood. Although surgical extraction is the standard treatment, its high recurrence rate and postoperative complications highlight the need for novel medical therapies [19, 20, 21]. Therefore, the underlying mechanisms of cholesteatoma require further study to identify new therapeutic targets, emphasizing the urgent need for research in this area.

Metabolomics analysis, an emerging technology, detects metabolites in biological samples for diagnostic and mechanistic research [22, 23, 24]. It has been used in cancer and infectious disease research [25, 26, 27], indicating its potential for studying CSOM with or without cholesteatoma. Metabolomics may reveal metabolic traits in patients with CSOM, aiding in the identification of new pathways and guiding the development of drug therapies. This study aimed to identify metabolites and pathways in CSOM patients by exploring metabolic changes from “safe ear” to “unsafe ear”. Two analysis techniques, ultra‐high performance liquid chromatography‐quadrupole time of flight‐mass spectrometry (UHPLC‐QTOF‐MS) and ultraperformance liquid chromatography‐tandem mass spectrometry (UPLC‐MS/MS), were employed to comprehensively cover tissue metabolomes in CSOM patients. These findings will contribute to understanding the pathophysiological mechanisms and inform prevention and treatment strategies for CSOM with cholesteatoma.

## 2 Materials and Methods

### 2.1 Study Population and Sample Collection

**FIGURE 1:**
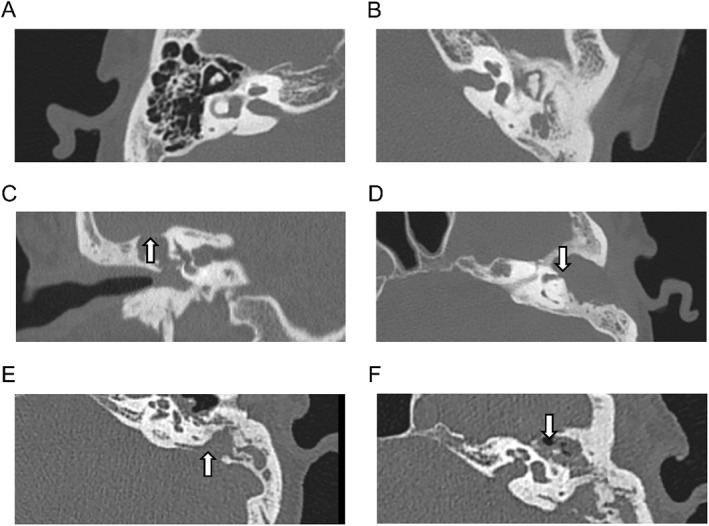
Radiological features of CSOM patients: (A) Normal ear, (B) patients from the CSOMP group: CSOM without cholesteatoma. MEC patients, which were classified as CT score 2 group, demonstrated the following CT scan findings (arrowed): (C) the tegmen tympani defect (arrowed), (D) the lateral semicircular canal defect (arrowed), (E) the sigmoid sinus bone plate eroded (arrowed), (F) the facial nerve canal eroded (arrowed).

This study was approved by the Ethics Committee of the Second Affiliated Hospital of Zhejiang University School of Medicine and conducted according to the Declaration of Helsinki. The relevant specimen collection process was reviewed and approved by the Ethics Committee of the Second Affiliated Hospital of Zhejiang University School of Medicine (approval number: 2020‐IRB‐182), and all enrolled patients signed relevant informed consent. From October 1, 2020 to January 1, 2023, patients with CSOM with or without cholesteatoma registered in the Department of Otolaryngology, the Second Affiliated Hospital of Zhejiang University School of Medicine, Hangzhou, Zhejiang, China. The demographic characteristics and clinicopathological data (age, sex, bone erosion range, etc.) of CSOM patients with or without cholesteatoma were saved for further analysis. Paraffin‐embedded tissue samples were collected from these patients for surgical pathological biopsies. Based on preoperative temporal bone CT scans, patients with cholesteatoma were classified into the MEC group, while those without cholesteatoma were classified into the CSOMP group as all of them were affected by tympanic perforation. The patients from the MEC group were then divided into two groups—CT score 1 and CT score 2, based on the degree of bone erosion present in the preoperative temporal bone CT scan [28]. Patients whose CT scan demonstrated a limited decline in scutum and/or auditory ossicles were scored 1. In contrast, those CT scans showing tegmen tympani erosion, lateral semicircular canal erosion, facial nerve canal erosion, and/or sigmoid sinus bone plate erosion were zoned to the CT score 2 (Figure 1). The other set of samples from 4 CSOM patients without cholesteatoma and 16 CSOM patients with cholesteatoma was collected as listed above. All tissue samples were obtained from patients during surgery, collected in a sterile container, immediately frozen in liquid nitrogen after cleaning, and stored at −80°C refrigerator until use. Our mass spectrometry‐based metabolomics approaches were conducted following the guidelines [22]. After surgery, all patients were monitored for postoperative recovery through routine clinical and imaging follow‐ups. The follow‐up protocol included imaging reexaminations conducted between 6 months and 1 year postoperatively to assess the healing of the surgical cavities and detect recurrence.

### 2.2 Sample Preparation

All metabolites in tissues from the screening set were extracted by the methanol method as follows: the tissues were weighed by analytical balance, and 100 μL HPLC methanol solution with 50% (methanol: H_2_O = 1:1, v/v) purity was added to each 20 mg tissue. After swirling at 4°C–8°C for 3 min and ultrasonication for 10 min, the samples were centrifuged at 13000 rpm for 10 min below 4°C. The supernatant was divided into two fractions for polar metabolites analysis by an Agilent 1290 Infinity II UHPLC system in tandem coupled to an Agilent 6545 Quadrupole Time‐of‐Flight mass spectrometry (UHPLC‐QTOF‐MS, Agilent Technologies, Palo Alto, CA, USA) system in either positive ion mode electrospray ionization (ESI) or negative ion mode ESI.

### 2.3 Global Metabolite Profiling Using UHPLC‐QTOF‐MS

The UHPLC‐QTOF‐MS system equipped with an ACQUITY UPLC BEH Amide column (100 mm × 2.1 mm, 1.7 μm) was used for the analysis. The mobile phase contained solvent A (15 mM ammonium acetate + 0.3% NH·H_2_O in water) and solvent B (15 mM ammonium acetate + 0.3% NH·H_2_O in 9:1 acetonitrile/water). The program for gradient elution was set as follows: 0–1 min, 95% phase B; 1–9 min, 95%–50% phase B; 9–12 min, 50% phase B; 12–12.5 min, 50%–95% phase B; 12.5–14 min, 95% phase B with a flow rate of 0.3 mL/min. The sample injection volume was 5 μL. MS data were acquired using ESI in positive and negative ion modes over 200–1200 m/z. Other main parameters for MS were set as follows: sheath gas temperature 300°C, sheath gas flow 11 L/min, VCap 3500 V, Capillary 0.09 mA, Nozzle voltage 0 V, gas temperature 275°C, fragment 150 V, and skimmer 65 V. The raw data of UHPLC‐QTOF‐MS were then imported into Profinder 10.0 (Agilent Technologies, Palo Alto, CA, USA) for peak detection, alignment, and integration.

### 2.4 Information of the Targeted Compound

The information of the targeted compounds this research used was as follows: azobenzene (CAS: 103‐33‐3, MCE), eicosapentaenoic acid ethyl ester (CAS:73310‐10‐8, MCE), kyotorphin (CAS:70904‐56‐2, MCE), marimastat (CAS:154039‐60‐8, MCE), and undecanedioic acid (CAS:1852‐04‐6, MCE).

### 2.5 Qualification of Targeted Metabolites Using UPLC‐MS/MS

As stated, the tissue samples from the validation set were resuspended in 100 μL 50% methanol solution. For differential metabolites analysis, UPLC‐MS/MS system with the SCIEX QTRAP 4500 triple quadrupole mass spectrometer (SCIEX, Framingham, MA, USA), coupled to a Nexera X2 LC‐AD detector (Shimadzu Corporation, Nakagyo‐ku, Japan) with an ESI interface and cooling autosampler was utilized. The main parameters for MS were set as follows: ESI, 500°C; the curtain gas, 25psi; nebulizer gas, ion spray voltage (IS), 5500 V/−4500 V; the spray gas and the auxiliary heating gas were 50 psi, respectively. The sample was injected into a Waters ACQUITY UPLC BEH C18 Column (2.1 mm × 50 mm, 1.7 μm) for quantitative analysis. The mobile phase consisted of solvent A (acetonitrile solution) and solvent B (aqueous solution containing 0.1% (v/v) formic acid). The program for gradient elution was set as: 0–1 min, 95% phase B; 1–4 min, 95%–0% phase B; 4–6 min, 0% phase B; 6–6.1 min, 0%–95% phase B; 6.1–10 min, 95% phase B with a flow rate of 0.2 mL/min. The sample injection volume was also 5 μL. Each analyte was performed in MRM parameters to determine the optimal conditions by fluidizing a single reference substance into an ESI source in either positive or negative ion mode. A gradient (0.5, 1, 2, 5, 10, 50, 100, 200, 500, 1000 ng/mL) was applied simultaneously to the temperatures of vaporizing. Sensitivity and specificity were optimized, and other parameters are shown in Table A1. Peak areas were used for calculations by Tracefinder 3.2 (Thermo, Carlsbad, CA, USA).

### 2.6 Data Acquisition and Analysis

The raw data were processed using Profinder 10.0 for peak detection, alignment, and integration. All of the metabolite variables were obtained from the UHPLC‐QTOF‐MS datasets. Principal component analysis (PCA), orthogonal projections to latent structures discriminant analysis (OPLS‐DA), and pathway analysis based on identified metabolites were performed using MetaboAnalyst 6.0 (http://www.metaboanalyst.ca) according to the Kyoto Encyclopedia of Genes and Genomes (KEGG) pathway database (www.genome.jp/kegg/). The differential metabolites were screened by the corrected *p* value and log_2_FC. The selection conditions were as follows: (1) log_2_FC > 1, and the difference threshold was 1, and (2) *p* < 0.05 was considered statistically significant. Data are presented as the medians (confidence interval, CI). *t*‐test and Mann‐Whitney *U* test were performed between the two groups. Chi‐square tests were used to compare categorical variables. Data were scaled to unit variance before conducting the PCA. Metabolite enrichment was log‐transformed before being subjected to pathway analysis.

## 3 Results

### 3.1 Characteristics of Study Patients

A total of 42 patients (mean [SD] age, 50 [14] years; 20 [48%] female) were divided into two groups for screening and validation. The anthropometric, clinical and biochemical parameters of the study patients from the screening and validation sets are summarized in Table 1 and in Table 2. There was no significant difference in age, gender, and other parameters between the CSOMP and the MEC groups in both sets. The demographic characteristics of the patients with different CT scores in the screening and validation sets, are presented in Table A2 and Table A3. Platelets in CT score 1 patients were significantly higher than those in CT score 2 patients from the validation set (*p* < 0.05). There was no significant difference in other parameters between the CT score 1 and the CT score 2 groups from both sets. Following surgery, all patients showed full recovery, with imaging results indicating successful healing of the surgical cavities, and no recurrence of the disease was observed during the follow‐up period between 6 months and 1 year postoperatively.

**TABLE 1:** Clinical characteristics of patients from screening set.

Variables		CSOMP (*n* = 3)		MEC (*n* = 19)		*p*
		Mean or medium	95% Confidence interval or (P25, P75)	Mean or medium	95% Confidence interval or (P25, P75)	
Age (years)		36.00	(−0.59, 72.59)	51.84	(45.07, 58.62)	0.788
WBC (10^9^/L)		5.50	(−1.36, 12.36)	6.40	(5.60, 7.20)	0.324
Neutrophil (10^9^/L)		3.23	(−2.09, 8.55)	3.71^a^	(2.93, 4.91)	0.787^b^
Lymphocyte (10^9^/L)		1.89	(0.64, 3.15)	1.96	(1.67, 2.24)	0.625
Platelets (10^9^/L)		168.67	(−23.60, 360.93)	234.53	(202.95, 266.11)	0.853
CRP (mg/L)		4.07	(−3.70, 11.83)	3.60^a^	(3.00, 5.20)	0.787^b^
Glucose (mmol/L)		4.71	(4.20, 5.21)	5.40	(5.03, 5.76)	0.233
Albumin (g/L)		45.10	(38.63, 51.57)	43.77	(41.99, 45.56)	0.504
Sex	Male (*n*)	1		10		0.534
	Female (*n*)	2		9		

Abbreviations: CRP, C‐reactive protein; WBC, white blood cell.

^a^ Not normally distributed, using median and P25‐P75 to display.

^b^ Mann‐Whitney *U* test.

**TABLE 2:** Clinical characteristics of patients from the validation set.

Variables		CSOMP (*n* = 4)		MEC (*n* = 16)		*p*
		Mean or medium	95% Confidence interval or (P25, P75)	Mean or medium	95% Confidence interval or (P25, P75)	
Age (years)		58.50	(41.34, 75.66)	46.47	(39.36, 53.57)	0.494
WBC (10^9^/L)		5.13	(3.82, 6.43)	6.21	(5.52, 6.90)	0.445
Neutrophil (10^9^/L)		2.93	(2.47, 3.38)	3.82	(3.25, 4.40)	0.083
Lymphocyte (10^9^/L)		1.68	(0.69, 2.67)	1.88	(1.61, 2.14)	0.487
Platelets (10^9^/L)		155.75	(98.23, 213.27)	230.47	(209.83, 251.10)	0.600
CRP (mg/L)		2.48	(−0.35, 5.30)	3.77	(2.61, 4.93)	0.707
Glucose (mmol/L)		5.45^a^	(4.91, 11.59)	5.13^a^	(4.84, 6.15)	0.335^b^
Albumin (g/L)		42.70	(40.63, 44.78)	44.12	(42.74, 45.50)	0.143
Sex	Male (*n*)	2		9		0.822
	Female (*n*)	2		7		

Abbreviations: CRP, C‐reactive protein; WBC, white blood cell.

^a^ Not normally distributed, using median and P25‐P75 to display.

^b^ Mann‐Whitney *U* test.

### 3.2 Non‐Targeted Metabolic Profiling of Tissue by UHPLC‐QTOF‐MS

**FIGURE 2:**
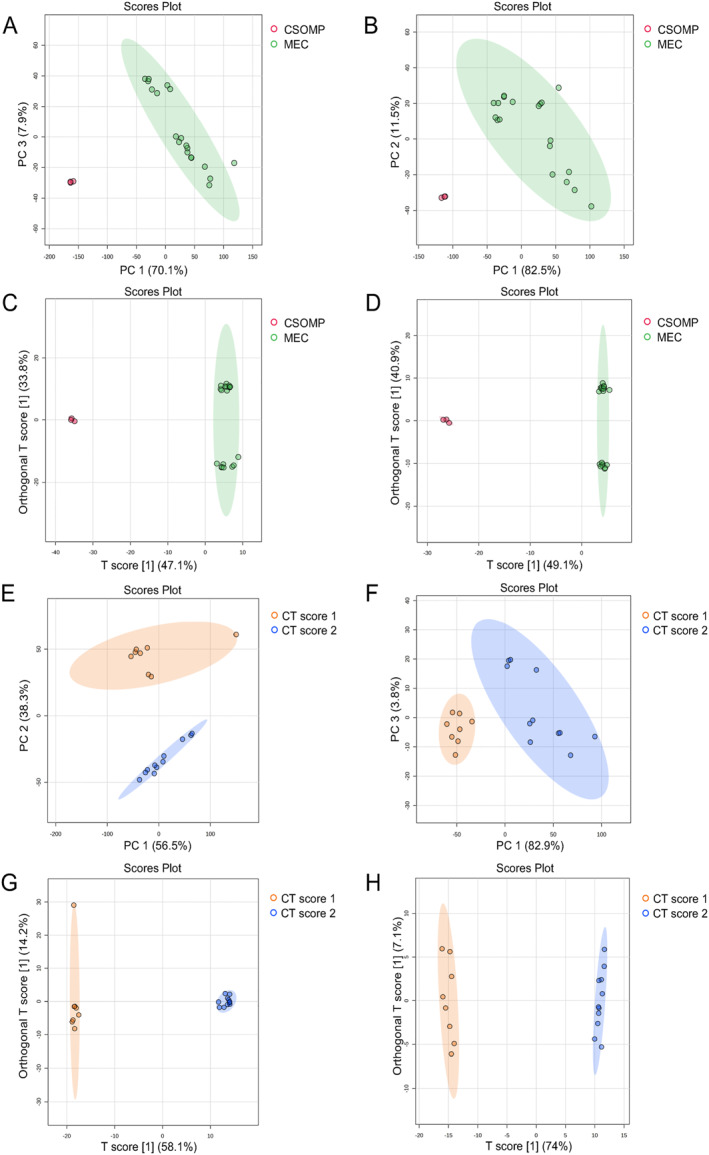
PCA and OPLS‐DA analysis plots from tissue sample metabolite spectra of positive and negative modes. The plots reveal clear separations of MEC versus CSOMP, and CT Score 1 versus Score 2. PCA analysis in (A) positive mode and (B) negative mode between MEC and CSOMP groups. OPLS‐DA analysis in (C) positive mode and (D) negative mode between MEC and CSOMP groups. PCA analysis in (E) positive mode and (F) negative mode between CT score 1 and CT score 2 groups. OPLS‐DA analysis in (G) positive mode and (H) negative mode between CT score 1 and CT score 2 groups.

**FIGURE A1:**
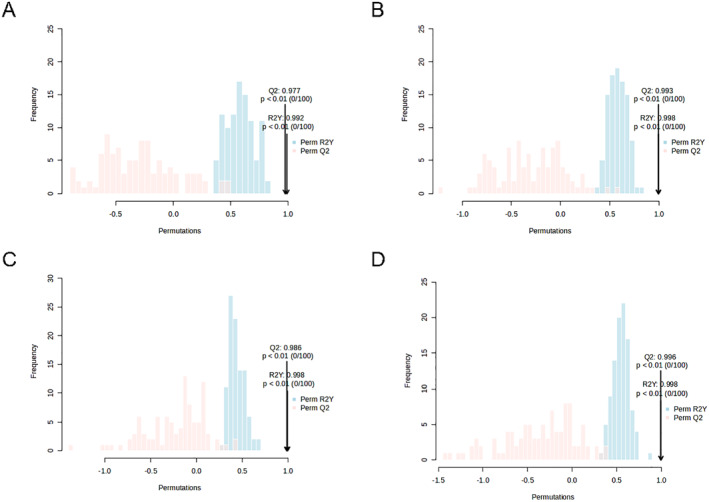
Statistical validation of the OPLS‐DA model by permutation test (100 times). (A) MEC and CSOMP (in positive mode); (B) MEC and CSOMP (in negative mode); (C) CT Score 1 and CT Score 2 (in positive mode); (D) CT Score 1 and CT Score 2 (in negative mode).

Non‐targeted metabolite profiling of the otitis media tissue samples by UHPLC‐QTOF‐MS was conducted to identify altered metabolites between the CSOMP group and the MEC group. The reproducibility of the metabolite features was assessed by coefficients of variation (CV), and those metabolites with significant variations (CV > 20%) were excluded prior to statistical analysis. After data annotation in the UHPLC‐QTOF‐MS dataset, 446 metabolite features were obtained in the positive mode and 234 in the negative mode. PCA and OPLS‐DA models were employed to assess whether the metabolic profiles of the MEC patients differed from those of the CSOMP patients and whether these differences were associated with the degree of bone erosion. An apparent separation trend was observed between the CSOMP and MEC groups in the PCA model for both positive (Figure 2A) and negative modes (Figure 2B), which was consistent with the trend seen in the OPLS‐DA score plots (Figure 2C,D). A clear separation trend was also observed between MEC CT score 1 and 2 groups (Figure 2E–H). Furthermore, the alignment verification of OPLS‐DA was carried out (*n* = 100, i.e., 100 permutation experiments were carried out). In the model verification, both R2Y and Q2 values were greater than 0.5, indicating that the model was stable and reliable (Figure A1). Moreover, all values of the intercept‐R2 and Q2 in the permutation test were lower than the original values (*p* < 0.01), which supported the validity of the OPLS‐DA models.

### 3.3 Altered Metabolites in Patients With Cholesteatoma

**FIGURE 3:**
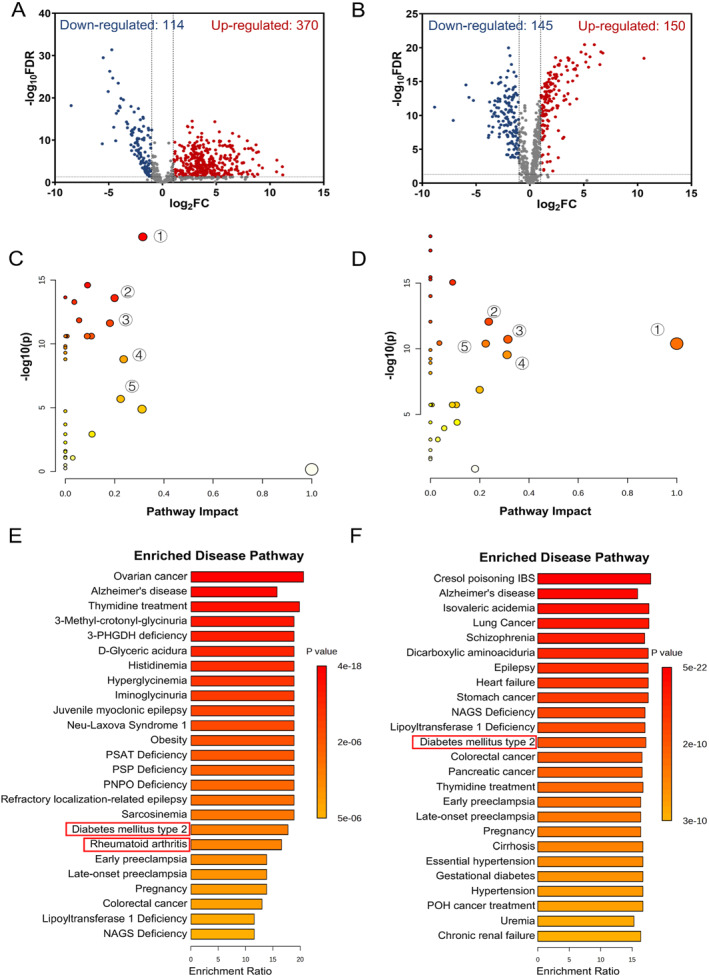
Volcano plot based on metabolites detected by the non‐targeted analysis: (A) MEC and CSOMP, (B) CT Score 1 and CT Score 2. The global pathway analysis of the metabolites: (C) MEC and CSOMP, (D) CT Score 1 and CT Score 2. Enriched disease pathways of the metabolites: (E) MEC and CSOMP, (F) CT Score 1 and CT Score 2.

Finally, different metabolites were screened to distinguish the MEC group from the CSOMP group, and the CT Score 1 group from the CT Score 2 group. A total of 370 significantly up‐ and 114 down‐altered metabolites were identified in the case of the MEC group versus the CSOMP group (Figure 3A). In the case of the CT score 2 MEC group versus the CT score 1 group, a total of 150 significantly up‐ and 145 down‐altered metabolites were identified (Figure 3B).

### 3.4 Differential Mapping of Metabolites in Pathway and Disease Analysis

**FIGURE 4:**
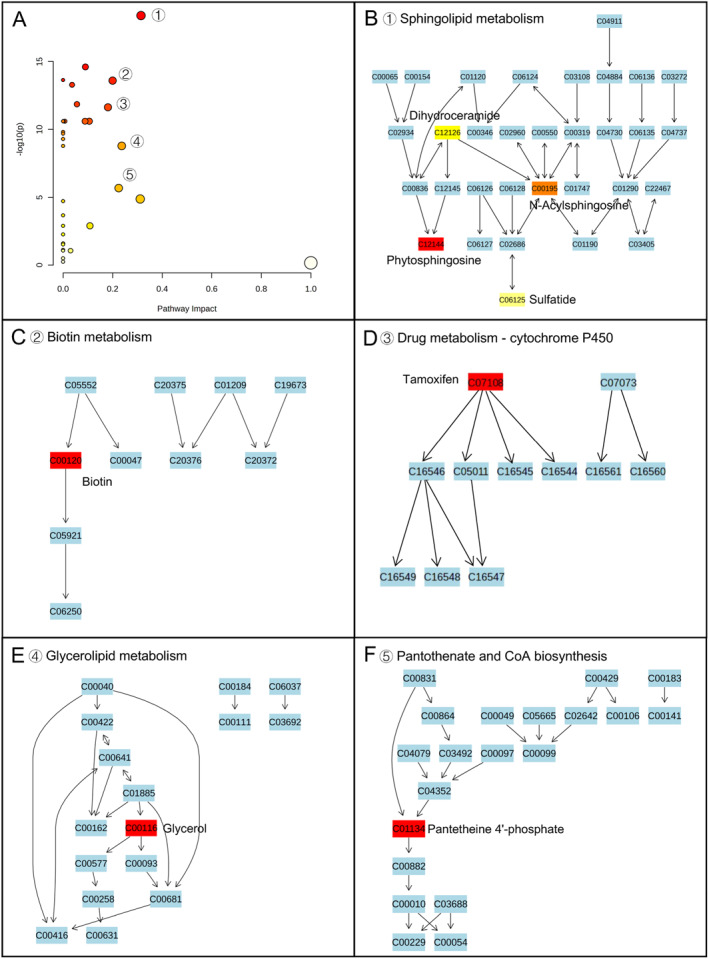
Global pathway analysis was performed using metaboanalyst: MEC and CSOMP. (A) Pathway enrichment analysis highlights the top enriched pathways between MEC and CSOMP: (B) sphingolipid metabolism, (C) biotin metabolism, (D) drug metabolism ‐ cytochrome P450, (E) glycerolipid metabolism, and (F) Pantothenate and CoA biosynthesis.

**FIGURE 5:**
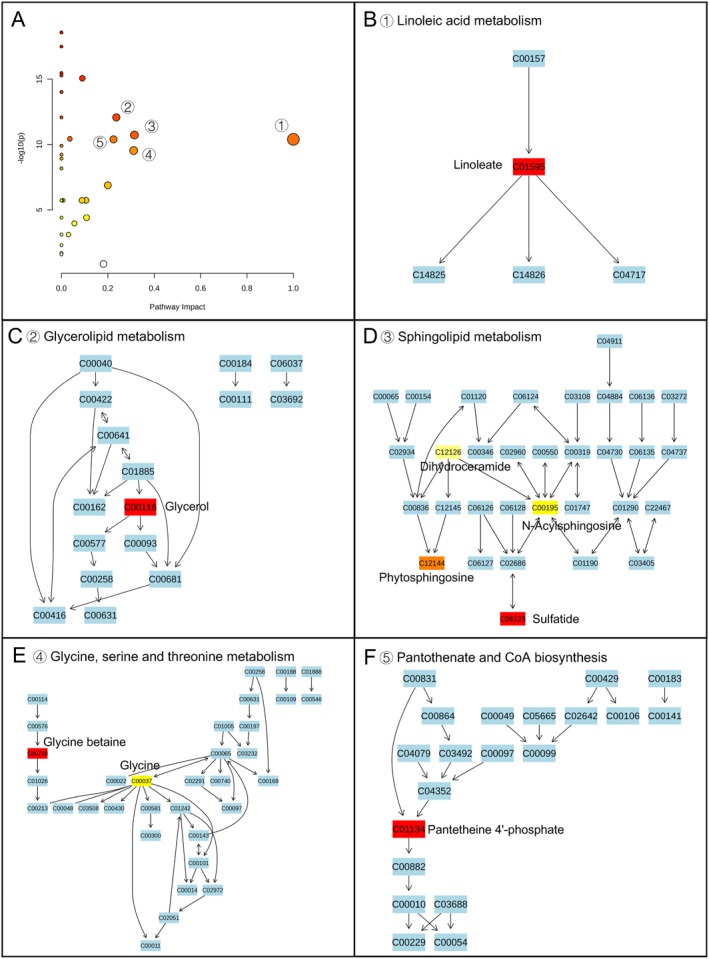
Global pathway analysis was performed using metaboanalyst: MEC CT Score 1 and CT Score 2. (A) Pathway enrichment analysis highlights the top enriched pathways between MEC CT score 1 and CT score 2 groups: (B) linoleic acid metabolism, (C) glycerolipid metabolism, (D) sphingolipid metabolism, (E) glycine, serine and threonine metabolism, and (F) pantothenate and CoA biosynthesis.

To explore the metabolic pathways that potentially contribute to the difference between MEC and CSOMP, we conducted a global metabolic pathway analysis (MetPA website: www.metaboanalyst.ca). By logarithmic transformation, a total of 32 pathways obtained were significantly different (false discovery rate (FDR) *p* < 0.05). As shown in Figure 3, small *p*‐values and large pathway‐impact values indicated highly influential pathways. Based on the impact values and *p* values (FDR *p* < 0.05; impact value > 0.2), the paths of sphingolipid metabolism, biotin metabolism, drug metabolism ‐ cytochrome P450, glycerolipid metabolism, and pantothenate and CoA biosynthesis were significantly altered in the case of MEC group versus CSOMP group (Figure 4). In the case of comparison of group CT score 2 and group CT score 1, the pathways of Linoleic metabolism, glycerolipid metabolism, sphingolipid metabolism, glycine, serine, and threonine metabolism, and Pantothenate and CoA biosynthesis were significantly altered (Figure 5). Disease enrichment analysis revealed that endocrine, metabolic and inflammatory‐related disorders were enriched (Figure 3E,F), such as rheumatoid arthritis and diabetes mellitus type 2. These results suggested that the primary variances between the MEC and CSOMP groups and between different CT scores were predominantly centered around Sphingolipid metabolism, biotin metabolism, glycerolipid metabolism and pantothenate and CoA biosynthesis pathway is considered a potential target in antimicrobial studies and immune dysregulation studies.

### 3.5 Metabolite Predictors of Future MEC Bone Erosion

We performed receiver operating characteristic curve (ROC) and area under the curve (AUC) analysis to globally visualize the significantly altered metabolites. Metabolites exhibiting significant tissue‐level patterns were identified as potential biomarkers for the early detection of MEC bone erosion. From these, we selected the top five metabolites: azobenzene, eicosapentaenoic acid ethyl ester, kyotorphin, marimastat, and undecanedioic acid (AUC = 1, log_2_(FC) > 5).

### 3.6 Qualification of Targeted Metabolites

**FIGURE 6:**
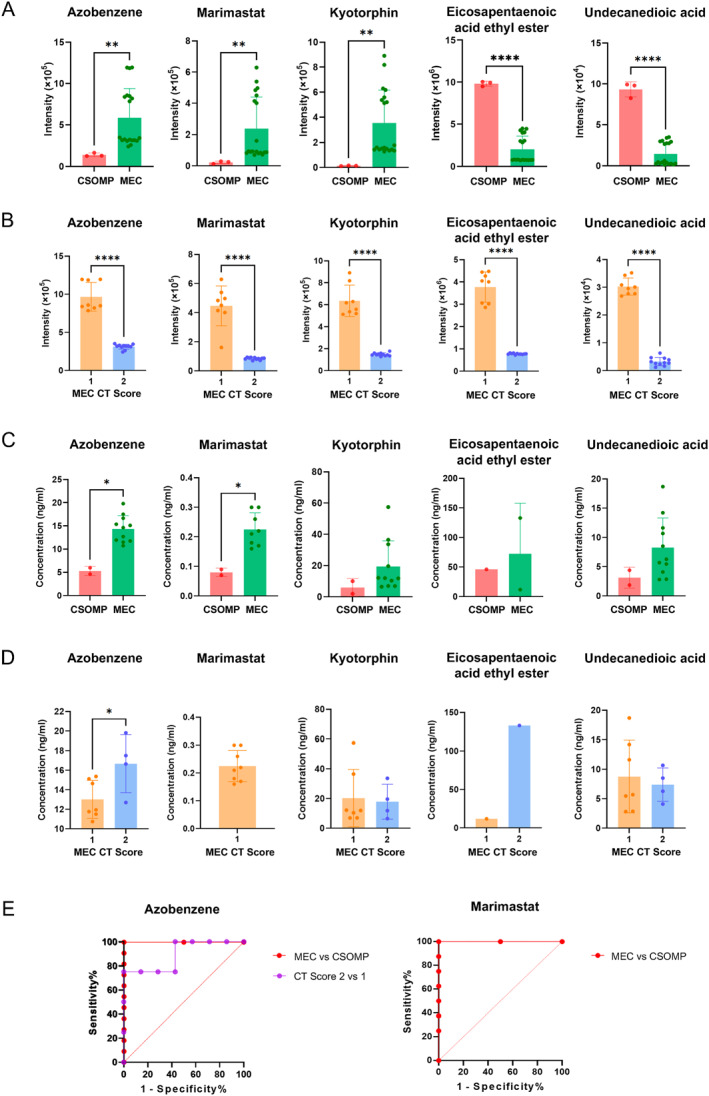
Multi‐platform metabolic profiling stratifies the progression of CSOMP and MEC. (A) The intensity of selected metabolites from the first cohort using non‐targeted platform: MEC and CSOMP. (B) The intensity of selected metabolites from the first cohort using non‐targeted platform: MEC CT Score 1 and CT Score 2. (C) The concentrations of selected metabolites from another independent cohort using targeted platforms: MEC and CSOMP. (D) The concentrations of selected metabolites from the other independent cohort using targeted platforms: MEC CT Score 1 and CT Score 2. (E) ROC analysis of two metabolites with differential levels: MEC versus CSOMP, and MEC CT Score 1 versus CT Score 2. *p < 0.05, **p < 0.01, ****p < 0.001.

**FIGURE A2:**
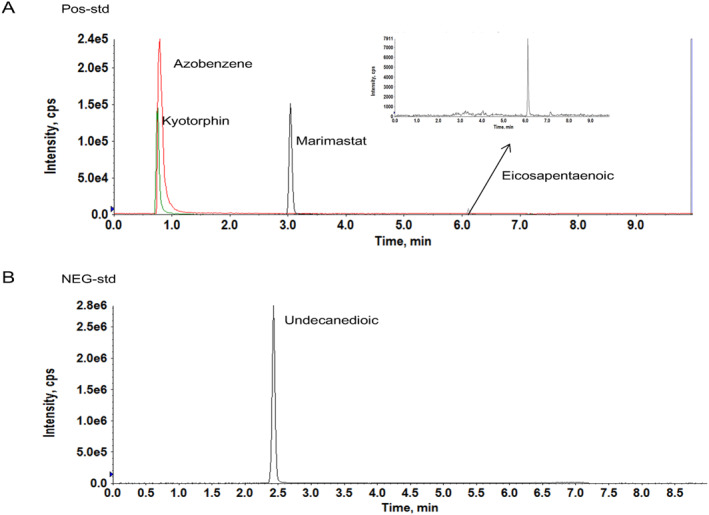
Extracted ion chromatograms of five standards in (A) positive mode and one standard in (B) negative mode.

**FIGURE A3:**
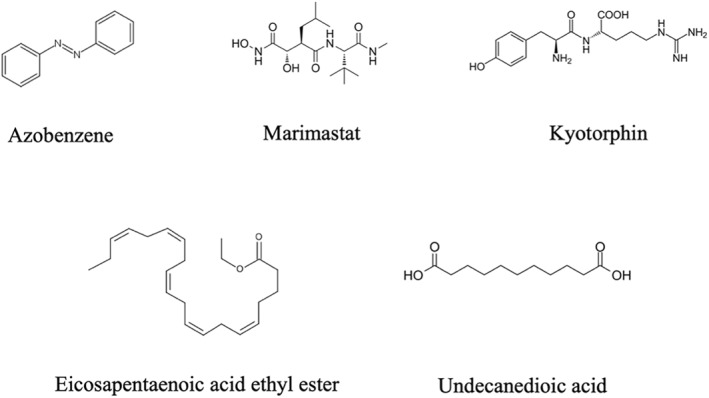
Structural formulas of targeted compounds.

To ensure that the level of differential metabolites is suitable for clinical application, we performed UPLC‐MS/MS to quantify these five metabolites in the other set (4 CSOMP, 16 MEC patients). Extracted ion chromatograms of four differential metabolite standards in positive mode and one standard in negative mode are shown in Figure A2. Structural formulas of targeted compounds are shown in Figure A3. Azobenzene and marimastat had higher intensity in MEC groups than in the CSOMP group (Figure 6A). It shared the same trend in the validation set: the level of azobenzene and marimastat was higher in the MEC than in the CSOMP group (*p* < 0.05, AUC > 0.7) (Figure 6C). However, kyotorphin, eicosapentaenoic acid ethyl ester, and undecane‐dioic acid, failed to have significantly different concentrations between the MEC and the CSOMP groups.

The ROC curves for azobenzene and marimastat are presented in Figure 6E, illustrating the relationship between clinical sensitivity and specificity across all possible cut‐offs. The ROC curves for both metabolites distinguishing MEC and CSOMP yielded an AUC of 1, demonstrating their potential for optimal diagnostic performance, with a best cut‐off providing 100% sensitivity and 100% specificity. However, given that these metabolites were extracted from tissue samples during surgery, the ROC curves and AUC analysis may primarily reflect their value in understanding the underlying pathophysiological mechanisms, rather than serving as diagnostic biomarkers. For further analysis, MEC cases were grouped based on the degree of bone erosion from their CT scans into two groups: CT score 1 and CT score 2. Azobenzene had lower intensity in MEC groups than in the CSOMP group (Figure 6B), while the concentration level was higher in the CT Score 2 than in the CT Score 1 (*p* < 0.05, AUC > 0.7) (Figure 6D). The AUC value suggests moderate discriminative power, which provides insight into its possible role in the pathological process.

## 4 Discussion

This study employed non‐targeted metabolomics to analyze tissue samples from CSOM patients with or without cholesteatoma, identifying metabolic differences across groups stratified by CT scores. Targeted metabolomics validation in an independent cohort verified five candidate metabolites: azobenzene, eicosapentaenoic acid ethyl ester, kyotorphin, marimastat, and undecanedioic acid.

Our findings revealed that 484 metabolites were significantly altered between the MEC and CSOMP groups in the non‐targeted metabolite profiling (Figure 3A). Based on these metabolic alterations, several pathways and diseases related to inflammation or bone erosion were enriched (Figure 3). Sphingolipid metabolism is closely associated with chronic inflammation in infectious diseases and inflammatory disorders [29, 30, 31, 32], and plays a vital role in the regulation of both trafficking and functions of immune cells [30]. Biotin is a water‐soluble vitamin synthesized de novo only by microorganisms, plants, and certain fungi, as this metabolic pathway is absent in humans [33, 34]. Consequently, biotin metabolism is considered as a novel therapeutic target for bacterial and fungal infections [34, 35]. Glycerolipid metabolism involves clearing reactive oxygen species (ROS) and modulating inflammatory‐associated signaling pathways in mucosa cells [36]. The pantothenate and CoA biosynthesis pathway is considered as a potential target in antimicrobial studies and immune dysregulation studies [37, 38]. These pathways demonstrated significant impacts on our analysis (Figure 3C,D). Disease enrichment analysis revealed that endocrine, metabolic and inflammatory‐related disorders, such as rheumatoid arthritis and diabetes mellitus type 2, were enriched (Figure 3E,F). Rheumatoid arthritis shares key molecules with MEC, leading to ectopic osteoclastogenesis and subsequent pathological bone destruction [15, 39]. Diabetes mellitus type 2 is associated with a characteristic pattern of dyslipidemia, in which similar cholesterols found in cholesteatoma are involved [40, 41].

After HCA and AUC analysis for 19 significantly altered compounds, five compounds—azobenzene, eicosapentaenoic acid ethyl ester, kyotorphin, marimastat, and undecanedioic acid, were marked for association with inflammation or transcriptional abnormalities. However, only azobenzene and marimastat levels showed significant differences between CSOM tissues with and without cholesteatoma in the validation set, mirroring the trends observed in the non‐targeted analysis (Figure 6).

Azobenzene is a photo‐switchable molecule widely used in industrial applications and biomedical research [42, 43]. This compound has been identified in human blood, but is not a naturally occurring metabolite [44]. The elevated azobenzene levels observed in MECs compared to CSOMP tissues may originate from environmental and occupational sources as part of the human exposome technically, and potentially initiate pathological cascades (Figure 6A,C). Azobenzene is reported to originate from azo dyes, some molecules of which have been reported to be murine carcinogens and enzymatically reduced to genotoxic aromatic amines with documented carcinogenic potential [45, 46]. Azobenzene itself is also reported to be a rat carcinogen, inducing invasive sarcoma [47, 48]. These findings suggest that azo dye exposure and azobenzene accumulation may drive the characteristic proliferative and invasive behaviors observed in MEC pathogenesis, showing its specific biological role in cholesteatoma formation.

Marimastat is a broad‐spectrum matrix inhibitor targeting matrix metalloproteinases (MMPs), including MMP‐2 and MMP‐9, demonstrating therapeutic potential for cholesteatoma‐related pathologies [49, 50]. Clinical investigations reveal that serum concentrations of MMP‐2 and MMP‐9 exhibit strong positive correlations with radiologically quantified bone erosion severity in both MEC and CSOMP patients [51]. MMP‐2 and MMP‐9 not only modulate inflammatory responses through pro‐inflammatory cytokine release in cholesteatoma microenvironments, but also have been characterized in histopathological specimens as a biomarker predictive of ossicular chain destruction [52]. Accumulating evidence indicates that disrupted equilibrium of MMPs and their tissue inhibitors drives progressive tissue degradation in ears with cholesteatoma [53, 54]. The accumulation of marimastat observed here may represent a compensatory feedback mechanism to delay bone erosion by inhibiting MMPs, though its long‐term efficacy requires evaluation in animal models. MMPs also participate in epithelial‐mesenchymal transition, the dysregulation of which contributes to pathological processes such as organ fibrosis, chronic inflammation, and cancer progression and metastasis [55]. Marimastat may suppress cholesteatoma ‐ related inflammation and invasion into surrounding tissues by blocking MMP‐dependent signaling pathways. The findings suggest marimastat's potential therapeutic utility in reducing MMP‐mediated inflammatory cascades during CSOM progression, particularly in cases exhibiting bone erosion of cholesteatoma.

Three metabolites (kyotorphin, eicosapentaenoic acid ethyl ester, and undecane‐dioic acid) failed validation using a targeted metabolomics platform due to multifactorial constraints. First, the technical difference between targeted and non‐targeted metabolomics played a predominant role. Based on the existing research, the detection threshold of these metabolites by the UPLC‐MS/MS system remains uncertain, limiting its clinical‐grade sensitivity. Ionization mode‐dependent quantification may cause biases between the two metabolomics platforms. Metabolites may show signal differences in two platforms with inconsistent ion modes, such as eicosapentaenoic acid ethyl ester, which was identified in the non‐targeted negative ion mode but quantified in the targeted positive mode. Second, sample processing methods may affect the quantification of these metabolites. The metabolites and their structural isomer interfere further quantification, as evidenced by the co‐elution pattern of undecane‐dioic acid and dodecanedioic acid. These observations highlight the critical need for stabilized collection protocols and dual‐platform verification in future studies.

The metabolites identified in this study show promise for clinical translation in the management of CSOM with cholesteatoma. These metabolites, with their different quantifications in diseases of varying severity, offer potential as diagnostic biomarkers that could be used to monitor disease progression or treatment response. Furthermore, both azobenzene and marimastat may be explored as therapeutic targets. Azobenzene's potential role in proliferative and invasive behaviors of cholesteatoma should be interrupted, and marimastat's impact on matrix degradation presents opportunities for targeted interventions in cholesteatoma management. Future studies should focus on validating these metabolites as clinical biomarkers and therapeutic targets, with further exploration of their specific molecular mechanisms in the pathogenesis of cholesteatoma.

All patients in this study fully recovered after surgery, and imaging performed between 6 months and 1 year postoperatively showed that their surgical cavities had healed well, with no recurrence of the disease observed. While this study focused on metabolomic profiling of surgically resected tissues, these initial clinical outcomes suggest that the biomarkers identified may have potential for monitoring postoperative recovery and predicting long‐term prognosis. Future studies with detailed postoperative follow‐up data could further explore the relationship between metabolic changes and recovery, thus enhancing the clinical utility of these biomarkers.

A key strength of this investigation is the combination of non‐targeted and targeted metabolomics approaches to identify biomarkers involved in cholesteatoma‐induced bone erosion, a mechanistically unexplored pathological process. However, three methodological limitations must be acknowledged: (1) While metabolite profiling was performed on surgically resected CSOM tissues (with/without cholesteatoma), our pathway enrichment analysis relied on systemic biofluids (serum/urine/stool), resulting in analytical discordance between tissue‐specific metabolic signatures and systemic biomarker interpretation frameworks. (2) The limited cohort size across in both the discovery (*n* = 21) and validation (*n* = 20) phases reduce the statistical power for definitive associations. (3) The sample size imbalance between the cholesteatoma (*n* = 35) and non‐cholesteatoma (*n* = 7) groups may affect statistical power and introduce bias. Clinically, the incidence of CSOM with cholesteatoma is significantly higher than that of CSOM without cholesteatoma requiring surgical resection, thus limiting sample availability. Several measures were taken during the biomarker screening process and statistical analysis, such as model robustness validation and non‐parameter tests to minimize the bias caused by small sample size and size imbalance. A study involving larger sampling and more balanced grouping is in progress. (4) The polymicrobial otitis microenvironment generates complex host‐microbe metabolic interactions that remain uncharacterized. Multicenter studies with standardized protocols and expanded sample size are required to validate these findings.

In conclusion, this metabolomics study identifies differential metabolite profiles and pathways between CSOM patients with and without cholesteatoma, and provides insight into the possible associations with inflammatory pathogenesis. Larger‐scale and longer‐term studies are required to validate these findings and elucidate the pathological mechanisms in CSOM patients with and without cholesteatoma.

## Author Contributions

**Lidan Hu:** conceptualization, methodology, formal analysis, writing – original draft, writing – review and editing. **Yifan Zhu:** formal analysis, visualization, writing – original draft. **Chengpeng Wu:** formal analysis, visualization, writing – original draft. **Xiao Liu:** investigation, data curation, writing – review and editing. **Qi Wang:** investigation, data curation, writing – review and editing. **Yangyiyi Huang:** investigation, data curation, writing – review and editing. **Hongyan Liu:** investigation, data curation, writing – review and editing. **Xiangjun Chen:** conceptualization, methodology, resources, project administration, writing – review and editing, supervision, funding acquisition. **Wei Wu:** conceptualization, methodology, software, formal analysis, writing – review and editing. **Hua Jiang:** conceptualization, methodology, resources, project administration, writing – review and editing, supervision, funding acquisition.

## Ethics Statement

This study was approved by the Ethics Committee of the Second Affiliated Hospital of Zhejiang University School of Medicine (SAHZU) and conducted according to the Declaration of Helsinki. The relevant specimen collection process was reviewed and approved by the Ethics Committee of the Second Affiliated Hospital of Zhejiang University School of Medicine (approval number: 2020‐IRB‐182), and all enrolled patients signed relevant informed consent.

## Consent

Informed consent was obtained from all subjects involved in the study. The authors affirm that human research participants provided informed consent for publication of the images in Figure 1.

## Conflicts of Interest

The authors declare no conflicts of interest.

## Data Availability

For privacy reasons, the data that support the findings of this study are available from the author, L. H., upon reasonable request.

## Appendix B Tables

**TABLE A1:** MRM table of the instrument.

Name	Q1	Q3	Dp	CE
Azobenzene	183	77	70	20
Marimastat	332.4	301.1	80	14
Kyotorphin	338.1	136	100	35
Eicosapentaenoic	348.4	285.3	30	10
Undecanedioic	215	197.1	−70	−23

**TABLE A2:** Clinical characteristics of CSOM with cholesteatoma patients from screening set.

Variables		CT score 1 (*n* = 8)		CT score 2(*n* = 11)		*p*
		Mean or medium	95% Confidence interval or (P25, P75)	Mean or medium	95% Confidence interval or (P25, P75)	
Age (years)		49.88	(36.05, 63.70)	53.27	(44.80, 61.67)	0.288
WBC (10^9^/L)		5.93	(4.43, 7.42)	6.75	(5.72, 7.77)	0.870
Neutrophil (10^9^/L)		3.06^a^	(2.62, 4.10)	4.26	(3.34, 5.17)	0.177^b^
Lymphocyte (10^9^/L)		1.89	(1.48, 2.30)	2.00	(1.54, 2.47)	0.147
Platelets (10^9^/L)		221.63	(161.22, 282.03)	243.91	(202.26, 285.56)	0.667
CRP (mg/L)		3.76	(3.03, 4.49)	4.40^a^	(2.80, 9.00)	0.717^b^
Glucose (mmol/L)		5.36	(5.01, 5.70)	5.42	(4.78, 6.06)	0.129
Albumin (g/L)		45.43	(43.64, 47.21)	42.57	(39.75, 45.40)	0.226
Sex	Male (*n*)	3		7		0.260
	Female (*n*)	5		4		

Abbreviations: CRP, C‐reactive protein; WBC, white blood cell.

^a^ Not normally distributed, using median and P25–P75 to display.

^b^ Mann‐Whitney *U* test.

**TABLE A3:** Clinical characteristics of CSOM in patients with cholesteatoma from validation set.

Variables		CT score 1 (*n* = 11)		CT score 2 (*n* = 5)		*p*
		Mean or medium	95% Confidence interval or (P25, P75)	Mean or medium	95% Confidence interval or (P25, P75)	
Age (years)		45.64	(36.65, 54.62)	48.75	(28.48, 69.02)	0.695
WBC (10^9^/L)		6.36	(5.45, 7.27)	5.8	(4.40, 7.21)	0.739
Neutrophil (10^9^/L)		3.85	(3.09, 4.61)	3.76	(2.44, 5.07)	0.907
Lymphocyte (10^9^/L)		1.98	(1.68, 2.29)	1.39^a^	(1.27, 2.14)	0.583^b^
Platelets (10^9^/L)		232.82	(215.36, 250.27)	224	(121.46, 326.54)	0.005^c^
CRP (mg/L)		4.06	(2.45, 5.68)	2.75^a^	(2.70, 3.48)	0.280^b^
Glucose (mmol/L)		5.04^a^	(4.84, 5.42)	6.38	(2.80, 9.96)	1.000^b^
Albumin (g/L)		44.3	(42.56, 46.04)	43.62	(39.70, 47.55)	0.983
Sex	Male (*n*)	6		3		0.838
	Female (*n*)	5		2		

Abbreviations: CRP, C‐reactive protein; WBC, white blood cell.

^a^ Not normally distributed, using median and P25–P75 to display.

^b^ Mann‐Whitney *U* test.

^c^ *p* < 0.05, significantly different between two groups.
